# Evaluation of PacBio Long-Read and PCR-Based Short-Read Sequencing for Mitochondrial DNA (mtDNA) Variant Detection, with an Emphasis on Detection and Quantification of mtDNA Deletion

**DOI:** 10.3390/ijms27083562

**Published:** 2026-04-16

**Authors:** Tanaya Jadhav, Matthew Aruta, Maria Alejandra Diaz-miranda, Avery Zucco, Laura K. Conlin, Ramakrishnan Rajagopalan, Jing Wang

**Affiliations:** 1 Division of Genomic Diagnostics, Department of Pathology and Laboratory Medicine, The Children’s Hospital of Philadelphia, Philadelphia, PA 19104, USA; jadhavt@chop.edu (T.J.);; 2 Department of Pathology and Laboratory Medicine, Perelman School of Medicine, University of Pennsylvania, Philadelphia, PA 19104, USA

**Keywords:** mitochondria DNA, mtDNA deletions, heteroplasmy, primary mitochondrial diseases, long-read sequencing

## Abstract

Accurate detection of all types of mitochondrial DNA (mtDNA) variants, including single large-scale mtDNA deletions (SLSMDs) and multiple mtDNA deletions (MMDs), along with heteroplasmy quantification, is essential for Primary Mitochondrial Disease (PMD) diagnosis. This study compares amplification-free PacBio long-read sequencing (LRS) mtDNA analysis with long-range PCR-based targeted mtDNA sequencing by short-read sequencing (SRS) in terms of detection sensitivity and accuracy. In total, 17 samples, including 4 SLSMD cases (3 blood, 1 muscle), 9 MMD muscle samples, and 4 deletion-negative controls (1 blood, 3 muscle), were sequenced using the PacBio Sequel IIe. Our findings demonstrate LRS’s efficacy in detecting single nucleotide variants (SNVs) and large mtDNA deletions with precise breakpoints. LRS can accurately detect and distinguish SLSMD from MMD, providing deletion heteroplasmy without the need for a second methodology. Deletion heteroplasmy computed from LRS was highly correlated with the Droplet Digital PCR (ddPCR) estimates (Pearson’s r2 = 0.95). While LRS can detect SNVs with approximately 5% heteroplasmy, only variants exceeding 10% heteroplasmy can attain 100% sensitivity, specificity, and precision when compared to those previously identified through clinical testing. In conclusion, our findings establish PacBio LRS as a robust tool for comprehensive mtDNA analysis capable of accurately detecting and quantifying heteroplasmic mtDNA variants and complex deletions.

## 1. Introduction

The mitochondrial genome is a 16,569 basepair circular DNA, densely packed with 13 protein coding genes that encode core subunits of the oxidative phosphorylation complexes, 22 RNA genes, 2 rRNA genes, and a non-coding control region (D-loop region) [1]. Primary mitochondrial disorders (PMDs) encompass a spectrum of highly heterogeneous disorders resulting from pathogenic variants in either nuclear DNA (nDNA) or mitochondrial DNA (mtDNA), affecting approximately 1 in 5000 individuals [2]. Due to the clinical heterogeneity of PMDs, a definitive molecular diagnosis is critical to confirm a PMD diagnosis. The mtDNA mutation spectrum includes single nucleotide variants (SNVs), small insertion/deletions (indels), single large-scale mtDNA deletions (SLSMD), and multiple mtDNA deletions (MMD), which contribute to the complexity of mtDNA testing. SLSMDs are deletions that span several kilobases in length, resulting in a wide phenotype spectrum with multisystemic involvement [3,4]. The most frequently observed large mtDNA deletion is a 4977 bp deletion occurring at chrM:8470-13447 (GRCh38 contig NC_012920.1) [5]. MMDs denote the presence of multiple large-scale mtDNA deletions of varying sizes co-occurring within a single sample. The presence of MMDs is often linked with deficiencies in nuclear genes responsible for mtDNA biogenesis and maintenance, as well as mitochondrial dynamics [6,7,8]. Although the exact mechanisms leading to large-scale mtDNA deletions remain elusive [8], it is crucial to distinguish MMD as a distinct entity from the syndromes caused by SLSMD. Due to the clinical and genetic heterogeneity of PMDs, a comprehensive analysis is essential to accurately identify mtDNA SNVs and structural variants to help solve undiagnosed cases and confirm PMDs.

It is well known that mtDNA deletion heteroplasmy levels are highly correlated with the onset and severity of symptoms observed in affected individuals [4]. However, the exact mechanisms underlying this correlation have yet to be fully established [3,8,9,10]. Accurate detection and differentiation between MMDs and SLSMDs alongside heteroplasmy quantification is essential for molecular diagnosis, clinical correlation, and disease prognosis.

Advanced sequencing technologies have evolved our understanding of mtDNA genetics, and in recent years, short-read next-generation sequencing (SRS)-based methods have been instrumental in revealing the rich diversity of mtDNA. Methods for clinical mitochondrial testing vary across clinical laboratories but often utilize targeted amplification of full-length mtDNA through long-range PCR (LR-PCR), followed by SRS to detect mtDNA deletions and SNV heteroplasmy [10]. Although the clinical SRS-based test exhibits sensitivity in detecting the presence of mtDNA deletions, it lacks the capability to quantify deletion heteroplasmy due to the inherent amplification bias introduced by LR-PCR. Therefore, a secondary assay such as Droplet Digital PCR (ddPCR) is required to quantify the large-scale deletion heteroplasmy levels. Neither method is effective in accurately detecting the deletion breakpoints, especially in cases with MMDs. Having precise deletion breakpoints is important to identifying the mitochondrial genes affected by the deletion and contributing to the clinical interpretation. Long-read sequencing (LRS) technologies such as PacBio HiFi sequencing have emerged as effective in addressing the shortcomings of short-read sequencing. In this study, we employed a PCR-free HiFi LRS combined with a custom bioinformatics workflow to accurately detect mtDNA SNVs and large-scale deletions. This approach allows us to accurately identify and differentiate mtDNA MMDs and SLSMDs, precisely determine breakpoints, as well as quantify deletion heteroplasmy, a capability that LR-PCR-based SRS lacks. We compared these values to the results from the standard-of-care mtDNA assay at our hospital [10] to determine the sensitivity and specificity of PacBio LRS and its potential as a comprehensive clinical test. A recent study by Lin et al. conducted a comparative performance analysis of Illumina-based SRS and PacBio LRS for mtDNA sequencing [11]. However, the experimental designs differed. In Lin’s study, LR-PCR products were utilized for both SRS and LRS, which inadvertently introduced amplification bias from the PCR-based assay. Furthermore, the study did not provide an evaluation of deletion quantification and did not to distinguish between MMD and SLSMD, which are crucial for accurate mtDNA-based diagnosis. Our study employs a distinct experimental design, analytical methodology, and clinical interpretation, thereby providing additional insights pertinent to clinical diagnostics.

## 2. Results

### 2.1. Coverage Analysis from LRS

PacBio reads aligned to the mitogenome showed even coverage with a median of 822× (range from 183× to 19,276×) (Table 1). The mean mtDNA coverage was 3292× (range from 233× to 19,276×) in muscle samples and 293× (range from 183× to 404×) in blood samples (Supplementary Tables S1 and S2). This coverage depth is consistent with published mtDNA content in these tissue types [12]. Of note, one muscle sample (Control 3, Table 1) exhibited exceptionally high average coverage of ~19,276×. Interestingly, this individual had a clinical test for mtDNA content of the same sample, showing mtDNA proliferation with a mtDNA content ~140% of age and tissue matched normal control, which is consistent with high coverage in LRS. SRS had a median coverage of 19,373× (range from 10,058× to 104,954×) with a mean coverage of 26,254× (range from 10,058× to 104,954×) in muscle samples and 71,463× (range from 40,940× to 89,601×) in blood samples. SRS had a much higher coverage than LRS due to using LR-PCR products for NGS library construction.

The mean nDNA coverage in blood was 14× (range from 10.5× to 16.9×), which was much higher than in muscle with mean coverage 2.1× (range from 0.2× to 9.3×) (Supplementary Tables S1 and S2). The low coverage of the nDNA in muscle samples is likely related to the high mtDNA content in muscle cells, which occupies a larger proportion of sequencing reads for mtDNA. An estimated mtDNA content was calculated based on mean coverage of mtDNA and nDNA in Supplementary Table S2.

**Figure 1:**
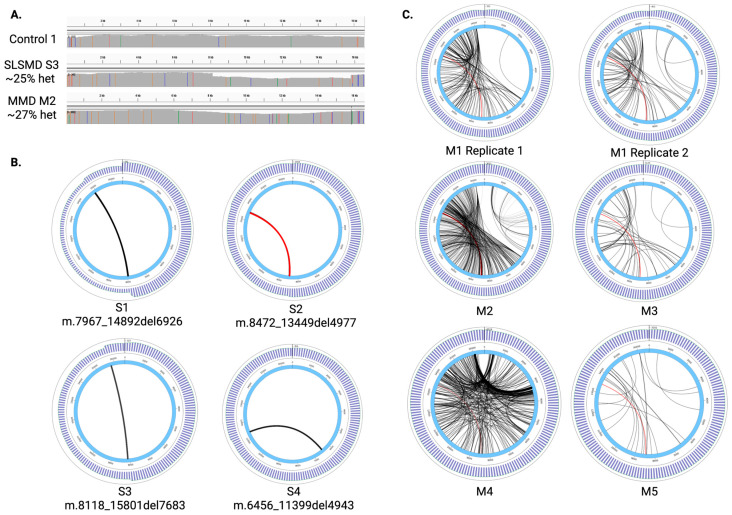
Mitochondrial DNA deletions in SLSMD and MMD samples. (**A**) Coverage across the mitogenome in a representative sample from each category—Control, SLSMD, and MMD. (**B**) Circos plots showing the single large-scale deletions observed in the SLSMD samples. The common 4977 bp deletion is highlighted in red. Thicker black lines represent deletion breakpoints seen at higher frequencies within the sample. The LRS coverage is shown in the outermost track. (**C**) Circos plots show the diversity of large-scale deletions observed in MMD samples. The common 4977 bp deletion is highlighted in red. The LRS coverage is shown in the outermost track.

SLSMD samples showed a sharp drop in coverage at the deletion breakpoints. MMD samples showed a gradual drop of coverage in SRS, but no such clear variation in coverage was observed across the mitogenome in LRS (Figure 1A).

### 2.2. Large-Scale mtDNA Deletion Detection and Heteroplasmy Quantification by PacBio LRS

PacBio LRS detected SLSMD in all four samples previously known to have SLSMD (Sample S1–4, Table 1). The deletion breakpoints are consistent with those previously reported by SRS (Figure 1B). The deletion heteroplasmy levels from LRS were consistent with those previously reported by ddPCR in all the SLSMD cases (Table 1). Sample S2 has the common 4977 bp mtDNA deletion [5,13], detected by both SRS and LRS (Figure 1B). Notably, the common 4977 bp deletion is the most prevalent deletion observed in sample M2 (Figure 1C, highlighted in red), consistent with previous reports that the accumulation of the recurrent 4977 bp mtDNA deletion has been observed in patients with muscular and neurological diseases, and linked with aging [14,15,16]. At least one molecule containing the common deletion was observed in every MMD sample.

Of the nine MMD samples, three samples (M7–9) exhibited extremely low deletion heteroplasmy. These samples had been previously confirmed using LR-PCR/SRS in clinical testing (Supplementary Figure S1), but their heteroplasmy levels were too low to be accurately quantified due to falling below ddPCR detection limits. Consequently, we reported their heteroplasmy levels as less than 10% (Table 1). Although the very low deletion heteroplasmy of these samples may also be below the detection limits of LRS, three deletion molecules were still observed in M8. Overall, LRS detected deletions in seven of the nine MMD samples. In samples where the ddPCR estimated heteroplasmy was in the 10–15% range, the discrepancy in heteroplasmy quantification by LRS was larger than in those with higher heteroplasmy (>15%). Only one molecule with a large deletion was observed in sample M6 using LRS despite having a mean coverage of 1461× due to its low deletion heteroplasmy (<10%) inferred from ddPCR quantification. The deletion heteroplasmy levels calculated from LRS and ddPCR had a Pearson’s correlation coefficient of 0.94 across all 13 SLSMD and MMD samples, 0.98 across the 5 SLSMD samples, and 0.83 across the 8 MMD samples (including the 2 replicates each of M1 and M7). In addition, Bland–Altman analysis was used to assess agreement between LRS and ddPCR heteroplasmy estimates in SLSMD and MMD samples (Supplementary Figure S2). In SLSMD samples, a consistent negative bias was observed (mean difference −9.60%), indicating systematic underestimation by LRS, with limits of agreement ranging from −19.37% to 0.18% (Supplementary Figure S2A). In contrast, MMD samples showed a smaller bias (−1.53%) but wider limits of agreement (−15.07% to 12.02%), reflecting greater variability (Supplementary Figure S2B). Differences in SLSMD samples were uniformly negative, whereas MMD samples exhibited both positive and negative deviations, indicating less consistent agreement. Overall, agreement between methods was stronger in SLSMD than in MMD samples, likely reflecting the heterogeneity of deletion breakpoints in MMD.

### 2.3. mtDNA SNV and Small Indel Analysis

SNVs and indels were called using both LoFreq and Mutect2 in the four control samples to assess their performance on PacBio LRS data (Table 2). Both variant callers showed high sensitivity, specificity, precision, and recall in samples Control 1–3. However, Mutect2 did not call the two SNVs in Control 4 (m.16098A>G and m.16320C>T). Based on manual inspection on IGV, both variants were real. The Mutect2 did not call both variants correctly due to an additional variant at the adjacent position (Supplementary Figure S3A). On the other hand, LoFreq failed to call two indels in Control 4 (m.513GCA>G and m.16188CT>C), which were located within or adjacent to a repeat region, and both were correctly detected by LRS (Supplementary Figure S3B). Overall, LoFreq only missed two indels that were called in SRS > 10% heteroplasmy and had no false positive calls, while Mutect2 missed two SNVs and called six false positive indels across all four samples. Due to the higher specificity and precision demonstrated by LoFreq in this data, we utilized it in our bioinformatics workflow and performed all further analysis using LoFreq variant calls.

To evaluate the accuracy and reproducibility of SNV detection using PacBio LRS, two samples (M1 and M7) were sequenced in replicate in different runs. The SNVs identified in two separate runs for each sample were 100% concordant in both M1 and M7 (Supplementary Table S3). A total of 14 SNVs were detected in both replicates of sample M1 using LRS, and 12 SNVs were detected in both replicates of sample M7. The SNVs detected in sample M1 are 100% consistent with the SRS data.

SNVs called from LRS and the two SRS runs (from two separate primer sets) for each sample are detailed in Supplementary Data—SNV.xlsx file. While the raw data from the LRS showed SNVs with low (<10%) heteroplasmy, the LoFreq variant caller (using the default settings) did not reliably call these variants in our workflow. Consequently, a low heteroplasmic variant of uncertain significance (VUS), *MT-TC* m.5791G>A at 6% heteroplasmy in Control 3, which was detected by SRS and LRS (Supplementary Figure S3C), but was missed by the LoFreq variant caller. In contrast, a 8% *MT-TE* m.14700C>T VUS in M7 was called by SRS but absent in LRS (Supplementary Figure S3D). After manual review on IGV, it appears that this variant is an artifact from SRS due to misaligned reads from multiple deletions in this sample. The LRS, on the other hand, perfectly avoided the misalignment and made accurate variant calls in this region. The sensitivity of SNV detection in our study is comparable to that reported by Lin et al. [11]. Both studies achieved a similar sensitivity of approximately 5%, despite using different SNV callers. While LoFreq missed a 6% m.5791G>A variant, it accurately called other low heteroplasmic variants, such as m.13897T>C at 4.7% (M7) and m.16327C>T at 6.1% (M6) (Supplementary Data).

Overall, all SNVs above 10% heteroplasmy showed 100% concordance in LRS and SRS variant calling and heteroplasmy levels. Neither SRS nor LRS detected any clinically significant small indels in this cohort. Due to this limitation in our workflow’s variant calling ability, only SNVs with >10% heteroplasmy in the two SRS datasets were used for our comparison. Overall, SNV detection from LRS yielded very high sensitivity, specificity, precision, and recall when compared to SRS calculated from the four control samples (Table 2). The SNVs with heteroplasmy < 10% may not be called using the default variant calling parameters in our workflow, and further parameter optimization is needed to call low heteroplasmy variants.

### 2.4. Impact of Read Depth and Coverage on Deletion Heteroplasmy

A downsampling experiment was conducted to determine the impact of read depth on the precision of deletion heteroplasmy calculation using LRS. Subsampled alignment files at varying depths were produced, and the deviation in heteroplasmy levels at different depths for each sample is shown in Supplementary Figure S4. The variation in calculated heteroplasmy levels was negligible (standard deviation ~0.013) at ≥500× mean coverage across the mitogenome. However, heteroplasmy levels were highly variable when mean coverage was <500×. The findings suggest that a sufficient read depth is necessary for accurate deletion heteroplasmy calculation in samples with low heteroplasmy. Samples M3, M4, and M5 represent low heteroplasmy samples (<10%) which show a very low probability of detection at coverage levels <500× with several replicates detecting <2% heteroplasmy. Coversely, in samples S1–4, which represent high heteroplasmy samples (25–65%), the probability of accurate heteroplasmy detection remains near 100% even at depths as low as 100×, because the signal-to-noise ratio is much higher. Although deletions are detected even at low coverage in high heteroplasmy samples, accuracy fluctuates significantly (5–10%) until a depth of at least 300× to 400× is reached.

### 2.5. Breakpoint Microhomology Analysis

**Figure 2:**
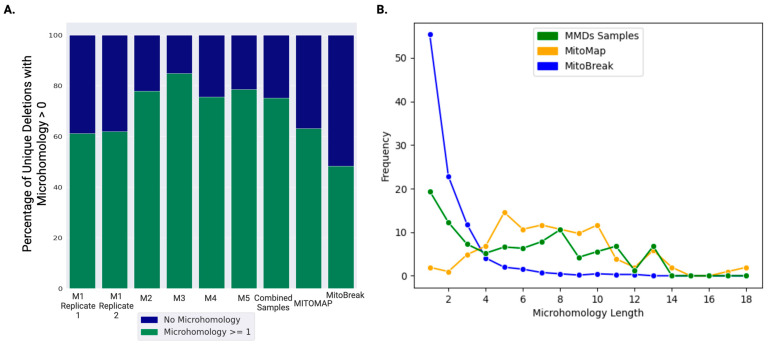
Analyzing microhomology at deletion breakpoints. Microhomology at breakpoints from five MMD samples (M1–5) was compared with deletion samples in MITOPMAP and MitoBreak databases. The “Combined Samples” are all deletions from the five MMD samples. The percentages from MITOMAP and MitoBreak are all reported deletion cases in these databases. (**A**) The percentage of deletions with microhomology ≥ 1 bp is shown in blue. The green bars are the percentage of deletions without microhomology. The replicate runs for M1 yielded the same results. (**B**) The frequency of microhomology lengths observed in the five MMD samples of our cohort is compared to the microhomology lengths in deletions from the MITOMAP and MitoBreak databases.

LRS has the distinct advantage of sequencing through the mtDNA molecules with large-scale deletions, thereby enabling the precise detection of the deletion breakpoints. The microhomology at deletion breakpoints was analyzed for MMD samples and compared with microhomology data from SLSMD in MITOMAP [17] and MitoBreak databases [18]. The percentage of deletions with microhomology ≥ 1 bp found at deletion breakpoints greatly varied per sample. Figure 2A shows the distribution of microhomology across five samples with sufficient deletions reads to support MMD (M1-M5, Table 1) and those reported in SLSMD samples from MITOMAP [17] and MitoBreak databases [18]. On average, 74.41% (standard deviation = 10.08) of deletions in each MMD sample had ≥1 bp microhomology at the breakpoints. Across all unique mtDNA deletions reported in MITOMAP and MitoBreak, the ≥1 bp microhomologies at the breakpoints are 63.19% are 48.3%, respectively. The number of bases of homology found at each deletion breakpoint ranged between 1 and 13 bp in the five MMD samples (M1–5) in this cohort but extended up to 18 bp in the deletions reported in the MITOMAP database and 14 bp in MitoBreak (Figure 2B). The frequency of deletions with homology lengths exceeding 10 basepairs observed in the MMD samples in this cohort is comparable to that observed in MITOMAP and MitoBreak. However, there were notable differences in the frequency of deletions with less than 4 bp of homology among MMD cases, MITOMAP, and MitoBreak. Interestingly, the frequency of deletions with less than 10 bp of homology in MMD samples was between MITOMAP and MitoBreak, both of which were from SLSMD (Figure 2B). This disparity in distribution may provide valuable insights into the diverse mechanisms underlying the formation of SLSMD and MMD.

## 3. Discussion

### 3.1. Overall Performance of PacBio LRS and SNV Detection

The need for an alternative mtDNA analysis approach arises from key limitations of the LR-PCR-based SRS method, which has been widely used in clinical diagnosis. While SRS provides high sequencing depth and supports sensitive detection of low-level heteroplasmy, its reliance on LR-PCR introduces amplification bias and cannot accurately quantify mtDNA deletion heteroplasmy. Moreover, although SRS performs well for detecting SNVs and small indels, its ability to comprehensively identify and characterize complex mtDNA structure variants and mtDNA duplications is limited. These limitations underscore the need for a single assay that can achieve both accurate structural variant detection and reliable heteroplasmy quantification in the clinical setting.

Amplification-free LRS addresses this gap by enabling the direct analysis of native mtDNA molecules without PCR bias. As demonstrated in this study, PacBio LRS achieves high accuracy in detecting single nucleotide variants (SNVs), small indels, and large-scale deletions, including short tandem repeat-mediated deletions (SLSMDs) and mitochondrial-mediated deletions (MMDs), when heteroplasmy levels exceed 10%. Notably, LRS allows for the accurate mapping of deletion breakpoints without confusion by microhomology at deletion junctions, and more comprehensive characterization of mtDNA structure variants. While sensitivity remains reduced for variants with low heteroplasmy, likely due to lower sequencing depth compared to SRS, further optimization of variant calling parameters may mitigate this limitation. SNV detection using PacBio LRS yielded high sensitivity, specificity, precision, and reproducibility when compared to SRS, demonstrating reliable detection for variants with heteroplasmy > 10%. However, we identified limitations in our workflow when calling SNVs with heteroplasmy below 10% threshold, as certain low-level variants were undetected by LoFreq using the default parameters at the available sequencing depth. This limitation suggests a possible need for parameter optimization of our variant calling workflow using LoFreq in specific cases where the detection of low-level heteroplasmic variants is essential to the clinical diagnosis. Collectively, LRS provides a complementary and potentially more comprehensive approach that addresses critical shortcomings of SRS and fulfills an unmet need in clinical mtDNA analysis.

NUMT interference should be considered when performing mtDNA analysis. A study has indicated that NUMT breakpoints were more common in the non-coding D-loop and mostly with sizes less than 1 kilobase [19]. Clinical tests for mtDNA analysis focus on the non-D-loop regions. The alignment pipeline used for LRS should effectively filter reads that contain potential NUMTs. The LR-PCR primer designed for SRS is specifically targeted for mtDNA and can avoid NUMT interference [20]. Both methods can effectively avoid NUMT interference and perform accurate mtDNA analysis.

### 3.2. mtDNA Large-Scale Deletion Heteroplasmy Quantification: LRS vs. ddPCR

We also compared mtDNA large-scale deletion heteroplasmy calculated by the number of deletion molecules detected by LRS vs quantified by ddPCR (Table 1). Comparisons with ddPCR and LRS showed a strong correlation in heteroplasmy quantification for SLSMD samples (Pearson’s correlation coefficient = 0.98), but lower in MMD samples (Pearson’s correlation coefficient = 0.83), which could be attributed to the limitations of using ddPCR for MMD heteroplasmy quantification. Bland–Altman Analysis of mtDNA deletion heteroplasmy in SLSMD and MMD showed similar results (Supplementary Figure S2). Similar deletion heteroplasmy discrepancies in low heteroplasmy samples have been reported in previous studies that used Nanopore LRS and qPCR [9]. MMDs are a group of large-scale mtDNA deletions with different sizes and breakpoints. The clinical ddPCR assay for deletion heteroplasmy quantification only targets two probe loci throughout the mitochondrial genome—one within the presumably non-deleted region (*MT-RNR2*), and the other within the common deletion region (*MT-ND4*). While this is true for SLSMD, breakpoints for MMDs can be highly variable. Some rare deletions in MMDs may encompass *MT-RNR2*, or not involve *MT-ND4*, which could result in inaccurate heteroplasmy results. Furthermore, the relative binding efficacies of the two ddPCR probes are not identical and may be altered by the presence of SNVs within the probe binding site in some samples. Since the heteroplasmy calculation from ddPCR relied on the relative abundance of the two probes, any variation in their binding efficacies affects the accuracy of the heteroplasmy calculation.

Nonetheless, the ddPCR assay has its limitations. Our data showed that LR-PCR and deep SRS coverage identified very low-level (<10%) large-scale mtDNA deletions, but these heteroplasmy levels could not be accurately quantified, or even detected, by ddPCR (Table 1 and Supplementary Figure S1). Consequently, the ddPCR assay lacked sufficient sensitivity to quantify these very low-level deletions. Similarly, other orthogonal methods for detecting mtDNA deletions, such as Southern blot, MLPA, and array CGH, lack high sensitivity and are unable to detect deletions below 20% [21] or 40% [22]. Based on current available technology, LR-PCR-based NGS is the most sensitive method for mtDNA deletion detection.

### 3.3. Advantages of LRS in Deletion Characterization

In recent years, several bioinformatics tools have been developed to enable the simultaneous detection of both mtDNA SNVs and large-scale deletions from short-read whole genome sequencing (WGS) data, including MitoSalt, MitoDel, and eKLIPse [23]. These approaches offer the advantage of integrating mtDNA analysis into routine WGS pipelines, potentially increasing diagnostic yield while reducing the need for separate targeted assays. However, limitations remain, particularly in accurately quantifying heteroplasmy levels of low-frequency deletions, especially in samples with low mtDNA content. While long-read sequencing offers improved resolution of structural features and more confident variant phasing, short-read WGS-based methods remain an attractive and scalable alternative for initial mtDNA screening in clinical and research settings.

Clustering PacBio LRS reads using DBSCAN enabled the differentiation between recurrent and unique deletions, a crucial aspect for diagnosing mitochondrial conditions that may involve multiple deletions of varying sizes. However, the clinical SRS assay faces limitations in precisely determining deletion breakpoints due to the frequent occurrence of microhomologies at breakpoint regions. Furthermore, it lacks the capability to quantify the frequency of each unique deletion in MMD samples. Analysis of breakpoint microhomology enabled by the precise deletion breakpoints from LRS could provide insights into the molecular mechanisms underlying mtDNA deletions [16]. The microhomology analysis in MMD samples revealed a pattern comparable to that reported in the MITOMAP database for SLSMDs. The 4977 bp deletion molecule, which has been reported as the most common deletion in SLSMD cases, was also most prevalent in MMD cases, indicating that the deletion formation in MMD and SLSMD may follow the same mechanism. Additionally, breakpoint analysis in MMD samples revealed homology lengths ranging from 1 to 13 bp, with the frequency of deletions with >10 bp of homology comparable to those reported in MITOMAP and MitoBreak. However, differences were observed in the frequency of shorter homology lengths (<4 bp) across datasets. While these variations may suggest differing mechanisms of deletion formation, such as replication slippage or non-homologous end joining [8], the data are insufficient to draw definitive conclusions at this time.

Although prior studies have proposed that MMD and SLSMD may arise through distinct molecular processes—potentially linked to chronic mtDNA replication stress in MMD versus single replication errors in SLSMD—the current analysis does not provide clear evidence to support this distinction. The overlap in homology patterns and the limited sample size constrain the ability to differentiate mechanistic origins with confidence. Further studies involving larger cohorts, functional validation, and high-resolution long-read sequencing are needed to clarify whether MMD and SLSMD truly reflect separate biological pathways or exist on a mechanistic continuum. Limited cohort size is an important constraint of this study. However, mitochondrial diseases are rare genetic disorders, and the prevalence of large-scale mitochondrial deletions is extremely low (estimated at 1.6/100,000 [24]), which hindered cohort selection.

PacBio LRS outperformed SRS in accurately annotating mtDNA deletion breakpoints and quantifying large-scale mtDNA deletion heteroplasmy levels without requiring a secondary methodology like ddPCR. While it can precisely detect SNVs, it has limitations in detecting large-scale mtDNA deletion below 10% heteroplasmy. A similar study compared mtDNA sequencing using Illumina SRS and PacBio LRS [11], which resulted in a SNV detection limit of 5% when Vardict v1.7.0 was used as the SNV caller. However, in Lin et al.’s study [11], both SRS and LRS were conducted using LR-PCR products, which inherit the amplification bias from LR-PCR and cannot accurately quantify mtDNA deletion heteroplasmy. In contrast, our study employed PacBio LRS utilizing total genomic DNA without PCR amplification, which enables precise quantification of mtDNA large deletion heteroplasmy. This approach facilitates comparison of the heteroplasmy quantification obtained using ddPCR.

### 3.4. Technical and Practical Limitations of LRS

Despite the advantages of PacBio long-read sequencing (LRS), several limitations were noted in this study. (1) The Sequel IIe system incurs a substantially high cost and low throughput compared with Illumina SRS. In this study, our focus is solely on analyzing mtDNA. It is important to note that over 99% of the total reads from the nuclear genome were excluded from this analysis, as the primary objective is to compare the performance of two methodologies for mtDNA testing. The current mtDNA clinical test employs LR-PCR to amplify the entire mitochondrial genome, followed by SRS [10]. Given that the test is specifically designed for mtDNA sequencing and utilizes LR-PCR products for NGS library construction, the regents cost per sample is much lower, varying depending on the number of samples batched per lane. Considering the current price, PacBio LRS is not feasible to serve as the first-tier clinical test for mtDNA analysis. However, it offers advantages in revealing complex structural variants, such as mtDNA duplications and complex structural variants, that are not detectable by SRS. Consequently, PacBio LRS is a valuable tool for unsolved cases with a suspected genetic etiology. (2) In this analysis, the sample utilized for PacBio LRS comprises total DNA, which includes both nDNA and mtDNA. The mtDNA content exhibits significant variability across different tissues, with an average coverage depth of 3172X in muscle and only 293X in blood (Table 1). Furthermore, there is intra-individual variability within the same tissue type. The low coverage depth observed in certain tissue types, such as blood, or in samples with compromised DNA quality or originating from individuals with mtDNA depletion syndromes, may potentially impact the test’s accuracy or potentially detect mtDNA variants at low heteroplasmy levels. The lowest average coverage from LRS was 183X in a deletion-negative control blood sample (as shown in S4 of Table 1). Despite this low coverage, LRS still achieved high concordance in SNV calling. However, there was a variant m.152T>C at 4.5% heteroplasmy that appeared to be missed by LoFreq but was visible in IGV (Supplementary Data—SNV.xlsx). (3) The sensitivity of LoFreq for detecting low-level heteroplasmic SNVs is limited, with a validated detection threshold of 10%. However, this analysis can detect SNVs below this level. Some SNVs previously detected by SRS at approximately 5% heteroplasmy might be missed by LoFrq caller but were visible in IGV. Therefore, we set our conservative threshold at 10% while working on improving the variant caller’s performance and increasing the sensitivity of SNV detection. Further improvements are necessary. (4) the detection of large-scale mtDNA deletions depends on the number of deletion-containing molecules captured. In certain cases, while mtDNA deletions were clearly detected by LR-PCR and SRS (Supplementary Figure S1), they were not observed by PacBio LRS, likely due to extreme low heteroplasmy (Supplementary Figure S5). Together, these findings highlight the current limitations of PacBio LRS in detecting variants at low heteroplasmy and underscore the need for further optimization of variant calling and sample preparation methods.

In summary, this study positions PacBio LRS as a promising technique for advancing mtDNA analysis, particularly in cases with complex deletion profiles or high clinical heterogeneity. PacBio LRS’s ability to detect mtDNA SNVs, small indels, and large-scale mitochondrial DNA deletions, quantify heteroplasmy, and precisely locate deletion breakpoints makes it a comprehensive tool for mtDNA analysis. By reducing the need for multiple assays and improving breakpoint accuracy, PacBio LRS could streamline mtDNA diagnostics and aid in accurate diagnosis of PMDs. However, to increase sensitivity for low heteroplasmy variants and further validate LRS’s utility in diverse clinical samples and mtDNA duplication detection, additional studies involving larger cohorts are recommended.

## 4. Methods

### 4.1. Cohort and Data Collection

This study conducted a technical evaluation of mtDNA sequencing results from SRS and LRS, using muscle and blood samples exhibiting SLSMD or MMD at varying heteroplasmy levels. The clinical correlation of these deletion samples has been previously studied [4,25]. In total, 17 samples—4 SLSMD samples (3 blood, 1 muscle), 9 MMD samples (muscle), and 4 deletion-negative controls (1 blood, 3 muscle)—from individuals who had previously undergone standard-of-care mtDNA testing by Illumina short-read sequencing (SRS) [10] and ddPCR at Children’s Hospital of Philadelphia (CHOP) were sequenced using the PacBio Sequel IIe (Table 1). Samples with potential complex structural variants, such as mtDNA duplications, were excluded from this study. Samples M1 and M7 were sequenced twice to assess reproducibility and the precision of heteroplasmy quantification. The SNV and deletion calls from PacBio LRS were compared with results from the clinical testing using SRS and ddPCR. All patients in this cohort consented to participate in research under a CHOP-approved IRB study protocol (#23-020790).

### 4.2. Long-Read Sequencing (LRS) and Bioinformatics Analysis

Total DNA, including nuclear and mitochondrial DNA, was extracted using a commercially available DNA isolation kit (PUREGENE DNA Isolation Kit, Qiagen, MD, USA). A total of 4500 ng DNA was diluted to 30 ng/μL, and sheared using either a Covaris G-tube or the Megaruptor 3 shearing kit. Sheared samples were prepared for sequencing using SMRTbell Library Prep Kit 3.0 and Sequel lle 3.2 binding kit, then sequenced on a PacBio Sequel lle system according to the manufacturer’s instructions (PacBio Inc. Menlo Park, CA, USA).

**Figure 3:**
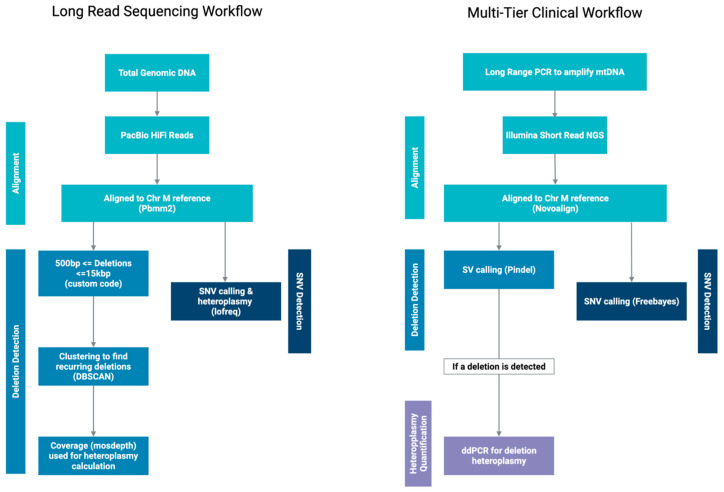
Bioinformatics workflow for LRS analysis compared to the multi-tier workflow used in clinical testing. The bioinformatics workflow used in this study to identify SNVs, SLSMDs, and MMDs from PacBio LRS is shown on the left. The bioinformatics workflow used by the clinical mitochondrial testing using SRS is shown on the right.

Each sample was sequenced using one SMRTcell on a PacBio Sequel lle system without any amplification, and the HiFi reads were aligned to the reference genome GRCh38 using pbmm2 v1.13.1with the iso-seq preset, which allows for lower gap extension penalties. The bioinformatics workflow used for large deletion and SNV calling is described in Figure 3. Reads aligning to the mitogenome (GRCh38 contig NC_012920.1) were extracted for further analysis. Large-scale deletions ranging from 500 bp to 15,000 bp in length were identified from the mitogenome alignment. Deletion breakpoints were clustered using DBSCAN [26] to identify recurrent deletions. Clusters containing more than one read with a deletion were considered recurrent. If all deletions in a sample were clustered together, the sample was classified as an SLSMD sample. Samples with multiple clusters were classified as MMD. Circos plots representing the deletion breakpoints were produced using PyCirclize [27]. Single nucleotide variants (SNVs) and small indels were called using LoFreq [28] and Mutect2.

### 4.3. Bioinformatics Analysis on Short-Read Next-Generation Sequencing (SRS)

All samples had previously been processed using the clinical testing workflow when they underwent comprehensive mtDNA testing. In brief, full-length mitochondrial DNA was amplified using LR-PCR using two sets of primers and sequenced using paired-end short reads on an Illumina HiSeq 2500 or NovaSeq 6000. Reads were aligned to NC_012920.1 using Novoalign. Deletions were called using Pindel [29] and SNVs were called using Freebayes Variant caller [30].

### 4.4. Deletion Heteroplasmy Quantification

Deletion heteroplasmy from LRS was calculated as the percentage of reads across the mitogenome that contained large-scale deletions. Since the clinical assay utilizes a probe located in the *MT-RNR2* gene to determine the total mtDNA molecules by ddPCR, the average coverage across the *MT-RNR2* gene was used to calculate the total number of mtDNA molecules sequenced. Samtools [31] was used to randomly subsample reads from all deletion samples, resulting in simulated datasets of varied coverage (between 5 and 100% of total coverage in 5% increments, 5 sub-replicates at each coverage). Deletion heteroplasmy was calculated for each dataset using the workflow described here and used to determine the standard deviation observed in the heteroplasmy level based on lower sequencing coverage.

Deletion heteroplasmy from SRS: when large-scale mtDNA deletions were detected by SRS-based clinical test, a follow up ddPCR assay on a Bio-Rad QX200 Droplet Digital PCR System using ddPCR Supermix for Probes (No dUTP) kit (#1863024, Bio-Rad, Hercules, CA, USA) will be performed to quantify the deletion heteroplasmy. In brief, two ddPCR reactions were carried out, one targeting the mtDNA commonly deleted region (MT-ND4) and the second targeting a region that is rarely involved in mtDNA large deletions (*MT-RNR2*). These two ddPCR reactions were used to determine the number of non-deleted mtDNA molecules and total mtDNA molecules, respectively. The deletion heteroplasmy level was calculated as [1 − (ND4/RNR2)] × 100% [10].

### 4.5. Deletion Breakpoints Microhomology Analysis

To evaluate sequence homologies at large-scale mtDNA deletion breakpoints, 20 basepairs (bp) in either direction of both 5′ and 3′ breakpoints were extracted for a total of 40 bp from each breakpoint. Microhomology length was defined as the length of perfect matches between the two 40 bp sequences. The distribution of unique deletions with microhomology and the length of homologous sequence observed in MITOMAP [17] and MitoBreak [18] were compared to MMD samples in our cohort. This analysis was performed with a subset of deletions without breakends in the D-loop region.

## Figures and Tables

**Table 1:** Sequencing coverage and heteroplasmy results for study cohort. The tissue type, mean sequencing coverage, number of deletions detected, and heteroplasmy from ddPCR as well as long-read sequencing for all samples is shown in this table. S1–S4: single large-scale mtDNA deletion (SLSMD); M1–M9: mtDNA multiple deletions (MMD); Control 1–4: deletion-negative samples. * <10% does not exclude 0% heteroplasmy. Samples M7 to M9 had a 0% deletion heteroplasmy as reported by ddPCR. However, the existence of large-scale deletions was confirmed in these samples by the SRS assay indicating that the deletions are present at very low-level heteroplasmy that is undetectable by ddPCR.

Sample ID	Tissue Type	Mean mtDNA Coverage	Number of Deletions Within 500–15,000 bp	Mean Coverage at *MT-RNR2* Gene	Heteroplasmy from LRS Using *MT-RNR2* Gene Coverage	Heteroplasmy from ddPCR	Deletion Type
S1	Blood	404.3	372	582.03	63.91%	73% ± 2	m.7967_14892del6926
S2	Muscle	2078.8	779	2330.24	33.43%	45% ± 2	m.8472_13449del4977
S3	Blood	265.9	80	316.84	25.25%	40% ± 4	m.8118_15801del7683
S4	Blood	319.3	107	344.87	31.03%	34% ± 0	m.6456_11399del4943
M1 Replicate 1	Muscle	451.2	120	487.52	24.61%	19% ± 4	MMD
M1 Replicate 2		346.2	67	414.11	16.18%		
M2	Muscle	3266.4	1097	3526.36	31.11%	15% ± 0	MMD
M3	Muscle	1047.9	41	1104.97	3.71%	12% ± 9	MMD
M4	Cardiac Muscle	6134.4	376	6299.55	5.97%	<10% *	MMD
M5	Muscle	3009.9	21	3032.39	0.69%	<10% *	MMD
M6	Muscle	1461.1	1	1549.59	0.06%	<10% *	MMD
M7 Replicate 1	Muscle	343.5	0	351.83	0.00%	<10% *	MMD
M7 Replicate 2		233.8	1	252.33	0.40%		
M8	Muscle	822.6	4	870.98	0.46%	<10% *	MMD
M9	Muscle	316.8	0	307.88	0.00%	<10% *	MMD
Control 1	Muscle	5058.9	0	5314.47	0.00%	Negative	Negative
Control 2	Muscle	5538.1	0	5925.83	0.00%	Negative	Negative
Control 3	Muscle	19,276.7	5	21,127.69	0.02%	Negative	Negative
Control 4	Blood	183.6	0	180.38	0.00%	Negative	Negative

**Table 2:** Comparison of SNV and small indel detection by LRS and SRS in 4 deletion-negative control samples using Mutect2 and LoFreq. TN: True Negative, the value is calculated for each sample by subtracting the total variants called from the total mtDNA length (16,569 bp); TP: True Positive, the value is the total number of SNV or small indels with heteroplasmy > 10%. FN: False Negative; FP: False Positive.

	Sample ID	LRS SNVs with Heteroplasmy > 10%	SRS SNVs with Heteroplasmy > 10%	Total mtDNA Length	TN	TP	FN	FP	Sensitivity (%)	Specificity (%)	Precision (%)	Recall (%)
SNV Detection using LoFreq	Control 1	16	16	16,569	16,553	16	0	0	100.00	100.00	100.00	100.00
	Control 2	37	37	16,569	16,532	37	0	0	100.00	100.00	100.00	100.00
	Control 3	15	15	16,569	16,554	15	0	0	100.00	100.00	100.00	100.00
	Control 4	34	34	16,569	16,535	34	0	0	100.00	100.00	100.00	100.00
SNV Detection using Mutect2	Control 1	16	16	16,569	16,553	16	0	0	100.00	100.00	100.00	100.00
	Control 2	37	37	16,569	16,532	37	0	0	100.00	100.00	100.00	100.00
	Control 3	15	15	16,569	16,554	15	0	0	100.00	100.00	100.00	100.00
	Control 4	32	34	16,569	16,535	34	2	0	94.44	100.00	100.00	94.44
Indel Detection using LoFreq	Control 1	0	0	16,569	16,569	0	0	0	0.00	100.00	0.00	0.00
	Control 2	1	1	16,569	16,568	1	0	0	100.00	100.00	100.00	100.00
	Control 3	1	1	16,569	16,568	1	0	0	100.00	100.00	100.00	100.00
	Control 4	0	2	16,569	16,567	2	2	0	50.00	100.00	100.00	50.00
Indel Detection using Mutect2	Control 1	2	0	16,569	16,569	0	0	2	0.00	99.99	0.00	0.00
	Control 2	2	1	16,569	16,568	1	0	1	100.00	99.99	50.00	100.00
	Control 3	2	1	16,569	16,568	1	0	1	100.00	99.99	50.00	100.00
	Control 4	4	2	16,569	16,567	2	0	2	100.00	99.99	50.00	100.00

## Data Availability

The original contributions presented in this study are included in the article/Supplementary Material. Further inquiries can be directed to the corresponding author(s). The bioinformatics workflow used in this study is available on github: https://github.com/tanaya-jadhav/MitoPac (accessed on 12 April 2026).

## Supplementary Materials

The following supporting information can be downloaded at: https://www.mdpi.com/article/10.3390/ijms27083562/s1.
