# Supplementary material for: Evaluation of PacBio Long-Read and PCR-Based Short-Read Sequencing for Mitochondrial DNA (mtDNA) Variant Detection, with an Emphasis on Detection and Quantification of mtDNA Deletion
Source: Int J Mol Sci. 2026 Apr 16;27(8):3562. doi: 10.3390/ijms27083562 (PMC13115627; doi:10.3390/ijms27083562)
Supplement: Supplementary file 1 [file ijms-27-03562-s001.zip › Supplementary Figures And Tables.pdf]

Supplementary Figure 1: Low level heteroplasmic MMD detected by long-range PCR and NGS.

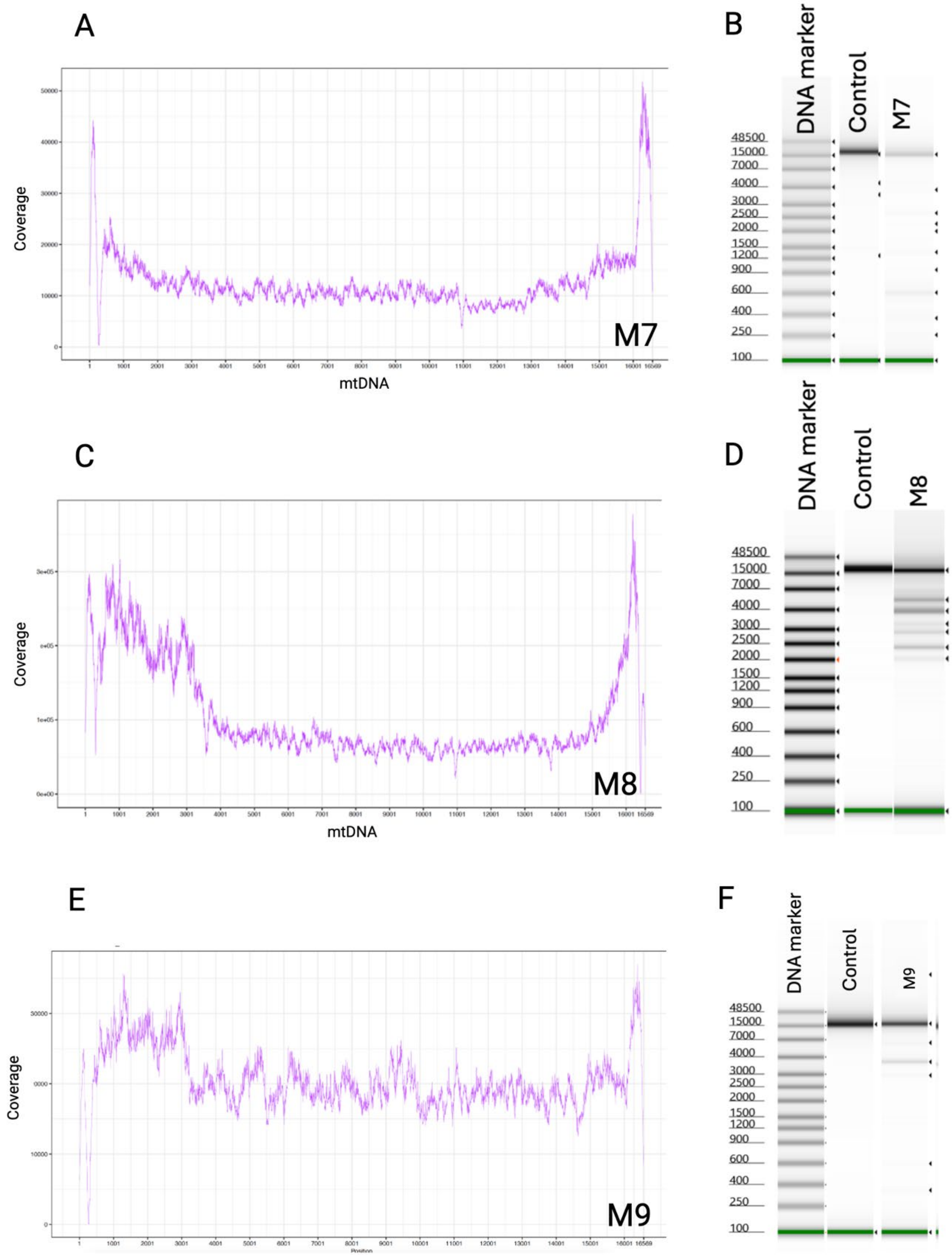

The NGS coverage profile showing the NGS read depth (y-axis) across the mtDNA position (x-axis) indicates the presence of mtDNA multiple deletions (A, C, E). Long-

range PCR gel images (B, D and F) showing multiple deletion bands support the detection of MMD by NGS. These gel images have been cropped to remove unrelated samples that were part of the same gel but are not relevant to this experiment. No other edits were made to the gel images.

Supplementary Figure 1G: Original, Uncropped Gel image for Sample M7

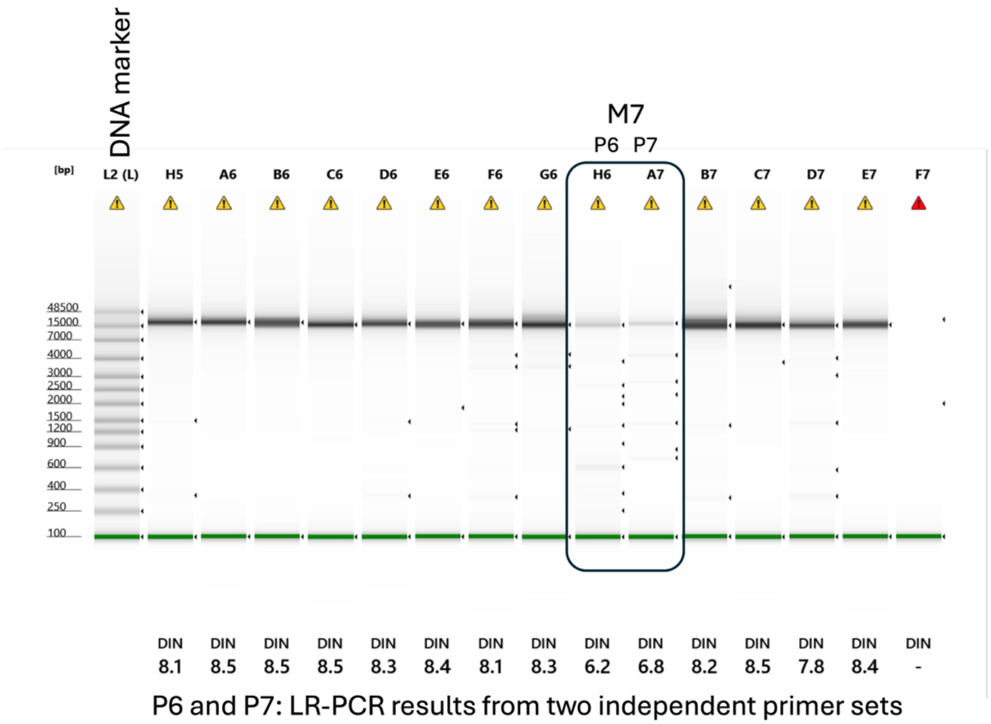

This figure shows the original, uncropped gel image containing sample M7. This MMD sample is shown in lanes H6 and A7. The labels P6 and P7 above each lane show the two independent primer sets used for this sample as part of the clinical testing protocol. Both lanes show bands of the full-length mitogenome at approximately 16.5 Kb as well as several fainter bands of varying molecular size showing mitogenome fragments containing deletions. This confirms the presence of a very low heteroplasmy deletions in sample M7

Supplementary Figure 1H: Original, Uncropped Gel image for Sample M8

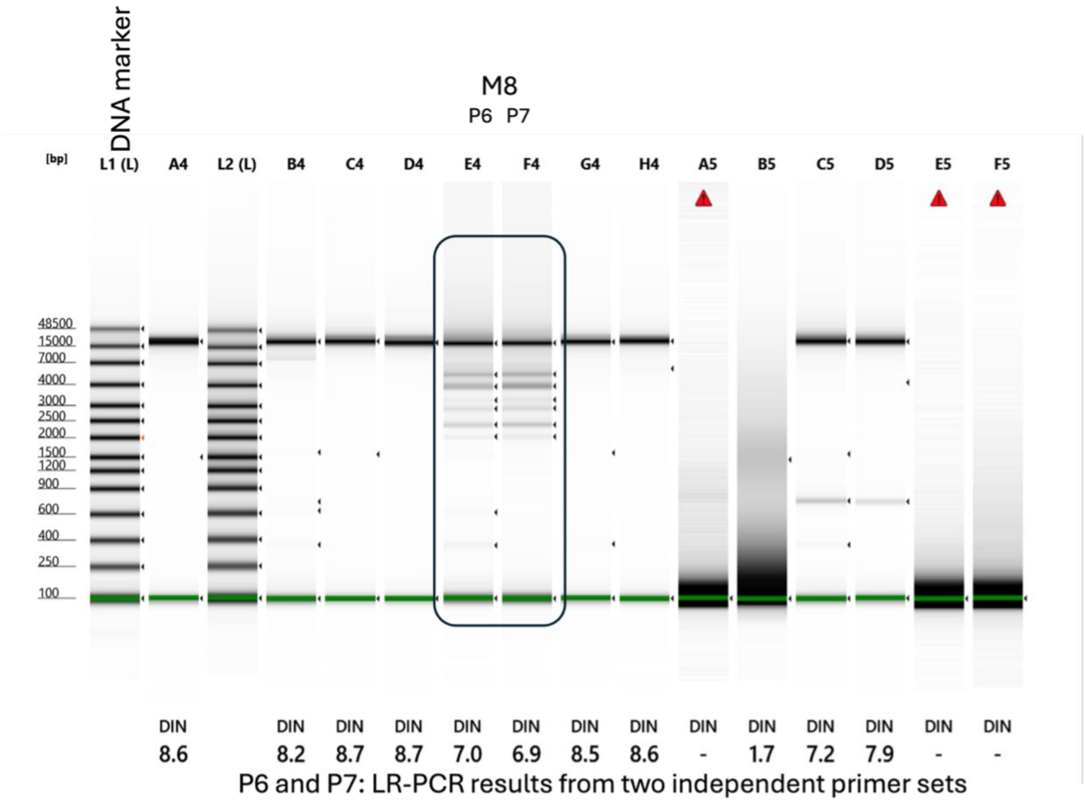

This figure shows the original, uncropped gel image containing sample M8. This MMD sample is shown in lanes E4 and F4. The labels P6 and P7 above each lane show the two independent primer sets used for this sample as part of the clinical testing protocol. Both lanes show bands of the full-length mitogenome at approximately 16.5 Kb as well as several fainter bands at of varying molecular size showing mitogenome fragments containing deletions. This confirms the presence of a very low heteroplasmy deletions in sample M8

Supplementary Figure 1I: Original, Uncropped Gel image for Sample M9

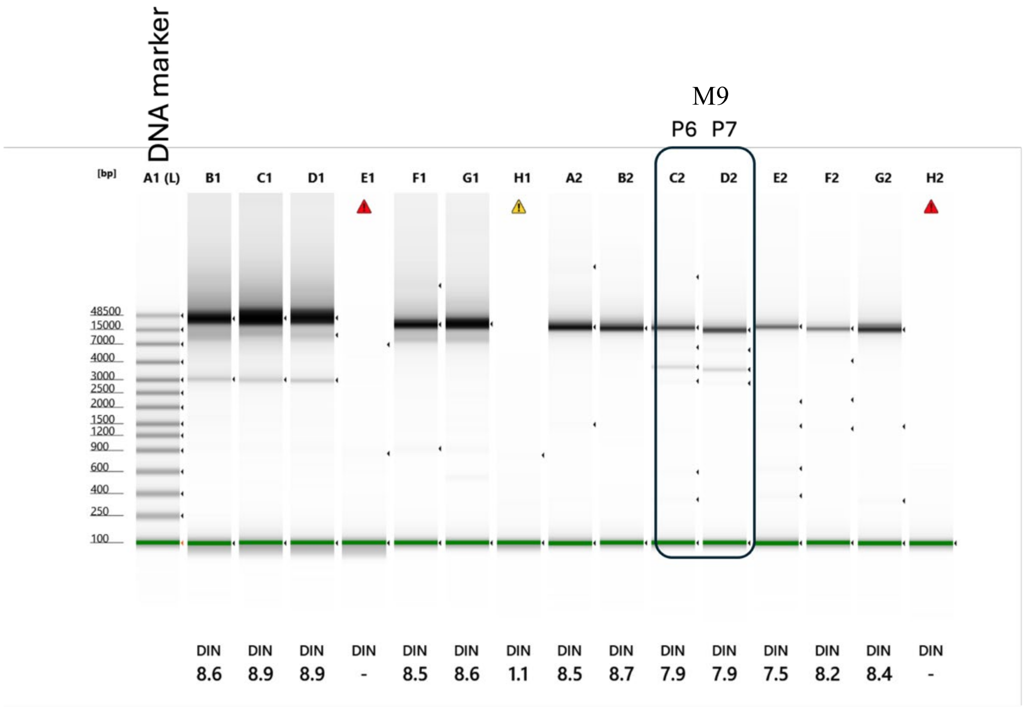

P6 and P7: LR-PCR results from two independent primer sets

This figure shows the original, uncropped gel image containing sample M9. This SLSMD sample is shown in lanes C2 and D2. The labels P6 and P7 above each lane show the two independent primer sets used for this sample as part of the clinical testing protocol. Both lanes show larger bands of the full-length mitogenome at approximately 16.5 Kb as well as several fainter bands of varying molecular size showing mitogenome fragments containing deletions. This confirms the presence of a very low heteroplasmy deletion in sample M9.

Supplementary Figure 2: Bland–Altman Analysis of mtDNA deletion heteroplasmy measurements by LRS and ddPCR in SLSMD and MMD Samples

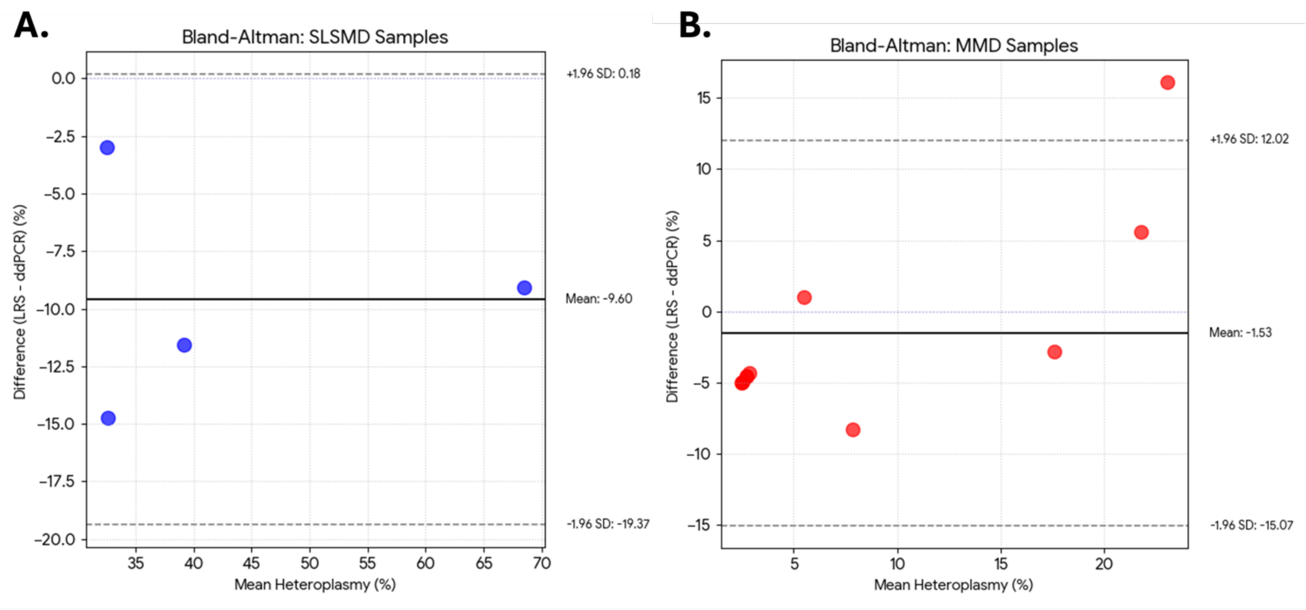

This figure shows plots for a Bland-Altman analysis for our cohort using the deletion heteroplasmy values determined from ddPCR testing and Long Read Sequencing data. A) The plot shows the difference between LRS and ddPCR heteroplasmy estimates (LRS − ddPCR) against their mean in 4 SLSMD samples. The solid line represents the mean difference (−9.60%), indicating a systematic underestimation of heteroplasmy by LRS. Dashed lines indicate the limits of agreement (−19.37% to 0.18%). All samples fall within these limits, with no apparent proportional bias across the range of heteroplasmy values. B) The plot shows the difference between LRS and ddPCR heteroplasmy estimates (LRS − ddPCR) plotted against their mean in all MMD samples. The solid line represents the mean difference (−1.53%), indicating minimal overall bias. Dashed lines indicate the limits of agreement (−15.07% to 12.02%), reflecting substantial variability between the two methods. Data points are distributed on both sides of zero, suggesting inconsistent agreement across samples and reduced concordance compared to SLSMD samples.

Supplementary Figure 3: Review of Discrepant SNV Variants in IGV

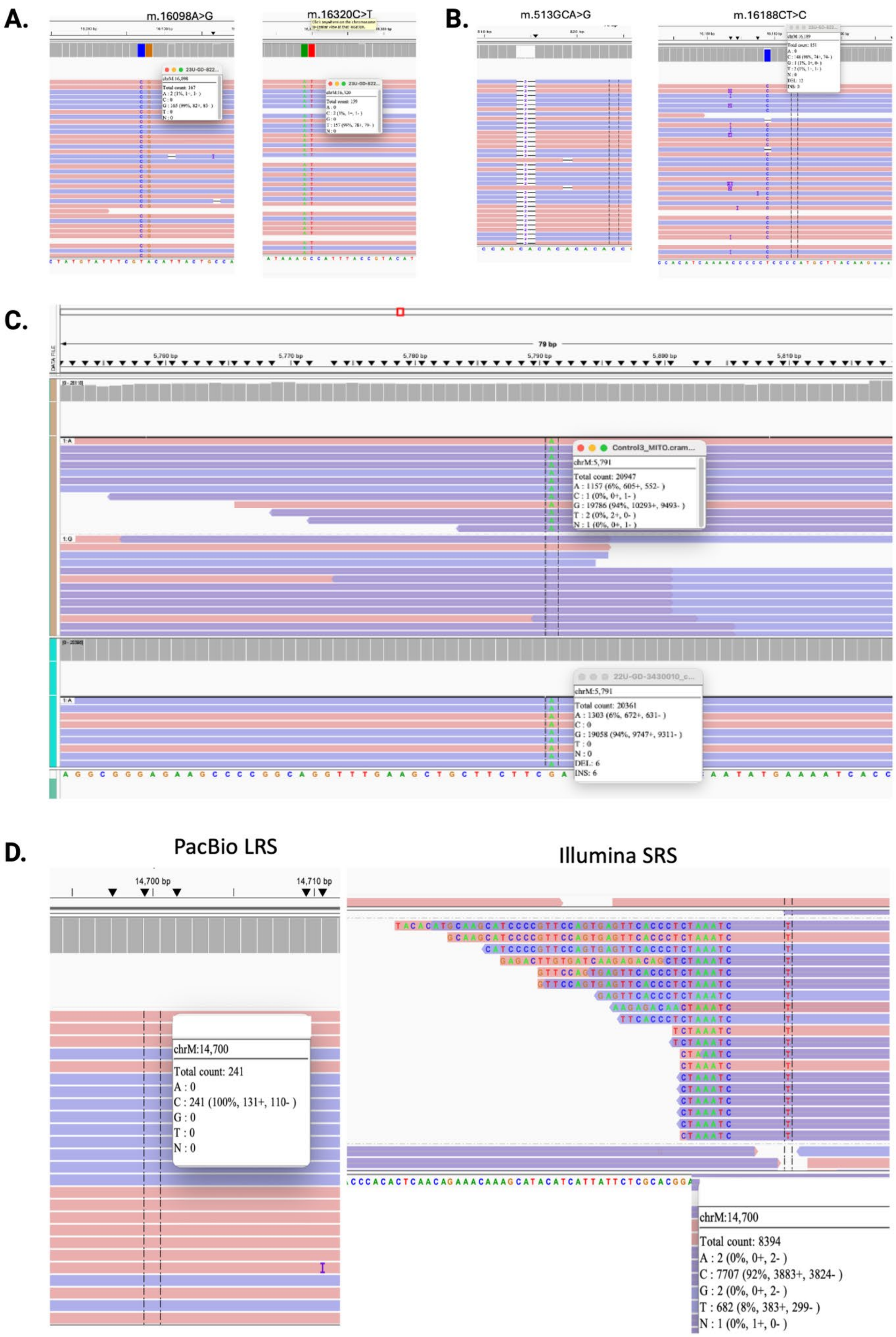
A.
m.16098A>G
m.16320C>T
B.
m.513GCA>G
m.16188CT>C
C.
79 bp
5,760 bp
5,770 bp
5,780 bp
5,790 bp
5,800 bp
5,810 bp
Control3_MITO.cram...
chrM:5,791
Total count: 20947
A : 1157 (6%, 605+, 552-)
C : 1 (0%, 0+, 1-)
G : 19786 (94%, 10293+, 9493-)
T : 2 (0%, 2+, 0-)
N : 1 (0%, 0+, 1-)
22U-GD-3430010_c...
chrM:5,791
Total count: 20361
A : 1303 (6%, 672+, 631-)
C : 0
G : 19058 (94%, 9747+, 9311-)
T : 0
N : 0
DEL: 6
INS: 6
D.
PacBio LRS
Illumina SRS
14,700 bp
14,710 bp
chrM:14,700
Total count: 241
A : 0
C : 241 (100%, 131+, 110-)
G : 0
T : 0
N : 0
chrM:14,700
Total count: 8394
A : 2 (0%, 0+, 2-)
C : 7707 (92%, 3883+, 3824-)
G : 2 (0%, 0+, 2-)
T : 682 (8%, 383+, 299-)
N : 1 (0%, 1+, 0-)

A) Mutect2 failed to call 2 SNVs (m.16098A>G and m.16320C>T) in Control 4, but both variants are real in IGV. B) LoFreq didn't call 2 Indels in Control 4 (m.513GCA>G and m.16188CT>C) that involving CA repeats and polyC track. C) A m.5791G>A variant was detected at 6% by both SRS and LRS, but was not called by LoFreq. D) The m.14700C>T variant from SRS in sample M7 is an artifact due to mismatched reads from multiple deletions.

Supplementary Figure 4: Effects of sequencing coverage on deletion heteroplasmy quantification.

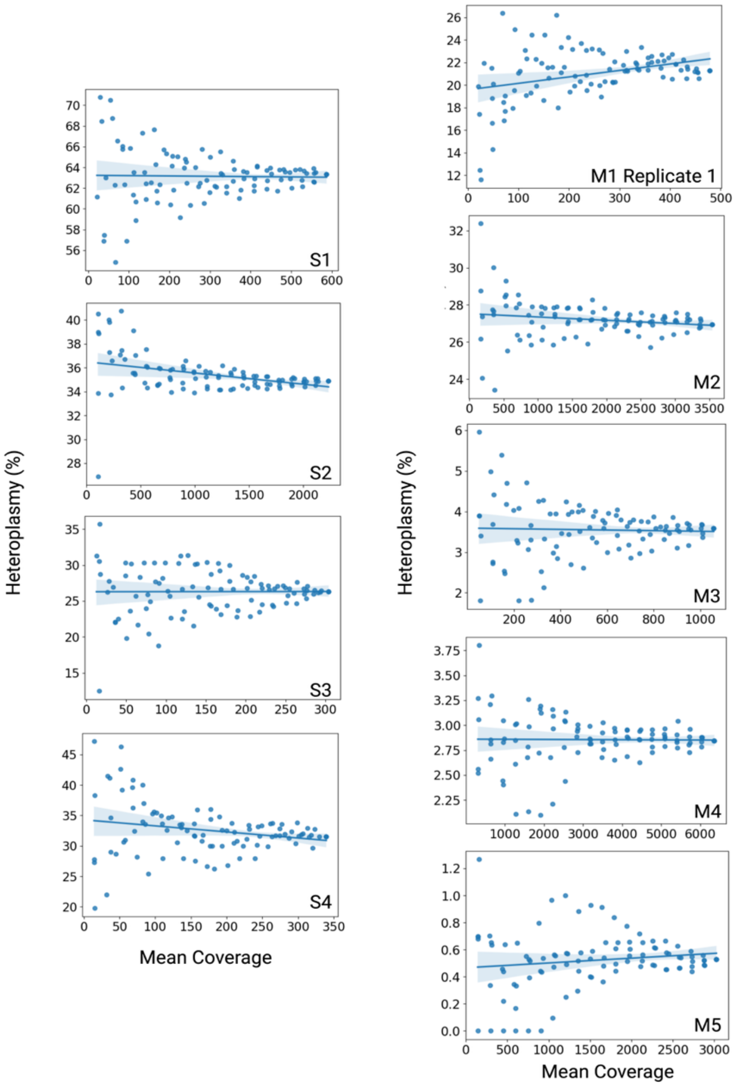

Datasets of varying mean coverage were simulated for each sample by randomly subsampling the long-read sequencing reads. Deletion heteroplasmy (y-axis) was calculated for each dataset and is plotted here against the mean coverage (x-axis).

Supplementary Figure 5: Coverage plots from Long-range PCR based short-read sequence (SRS) and PacBio long-read sequence (LRS). X axis: mtDNA positions; Y axis: coverage depth.

A. **Coverage Plots for SLSMD samples**

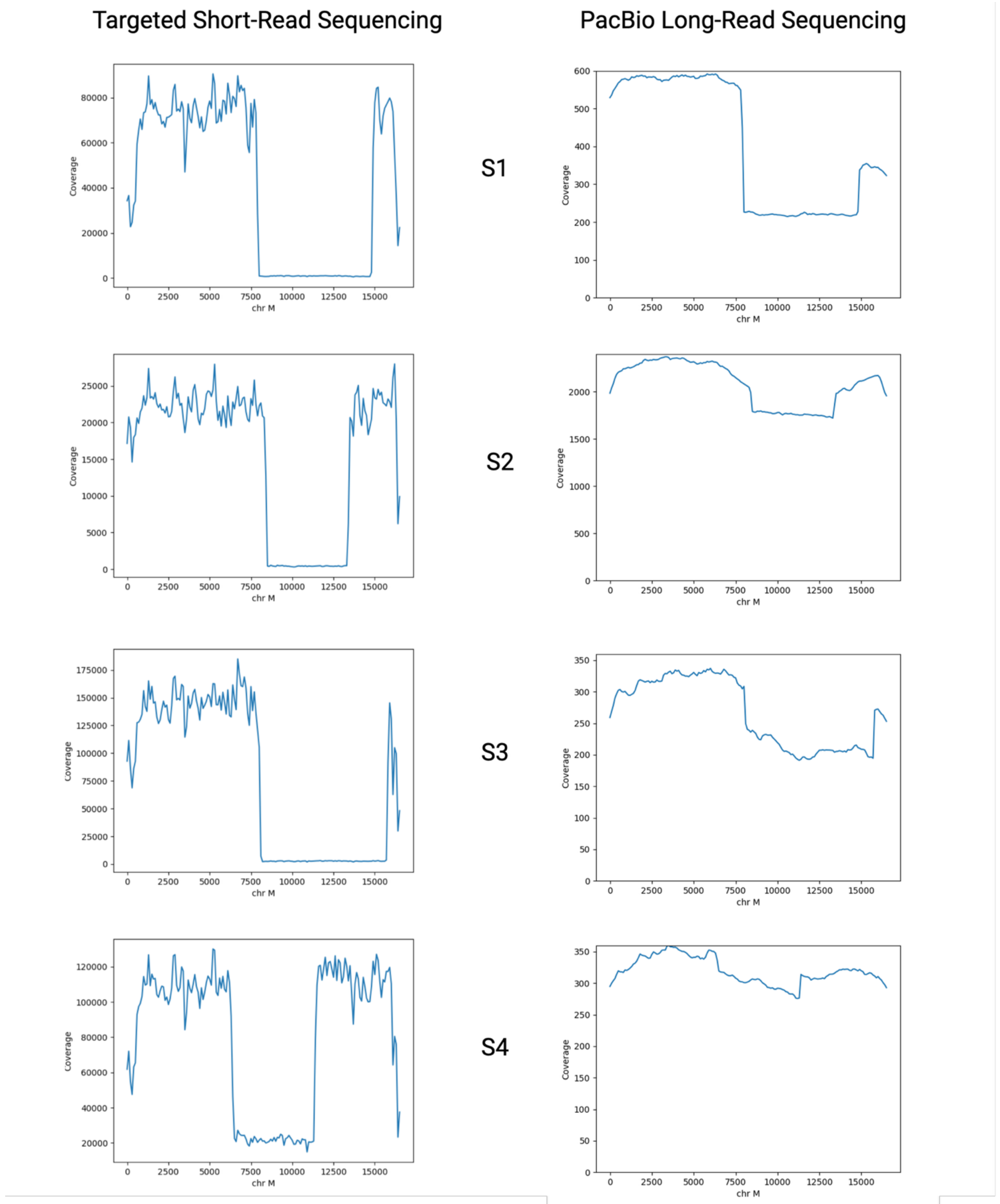

This figure plots coverage for SRS and LRS data across the mitogenome for SLSMD samples. SLSMD samples show drops in coverage at the breakpoints of the large mtDNA deletions.

B: Coverage Plots for MMD Samples

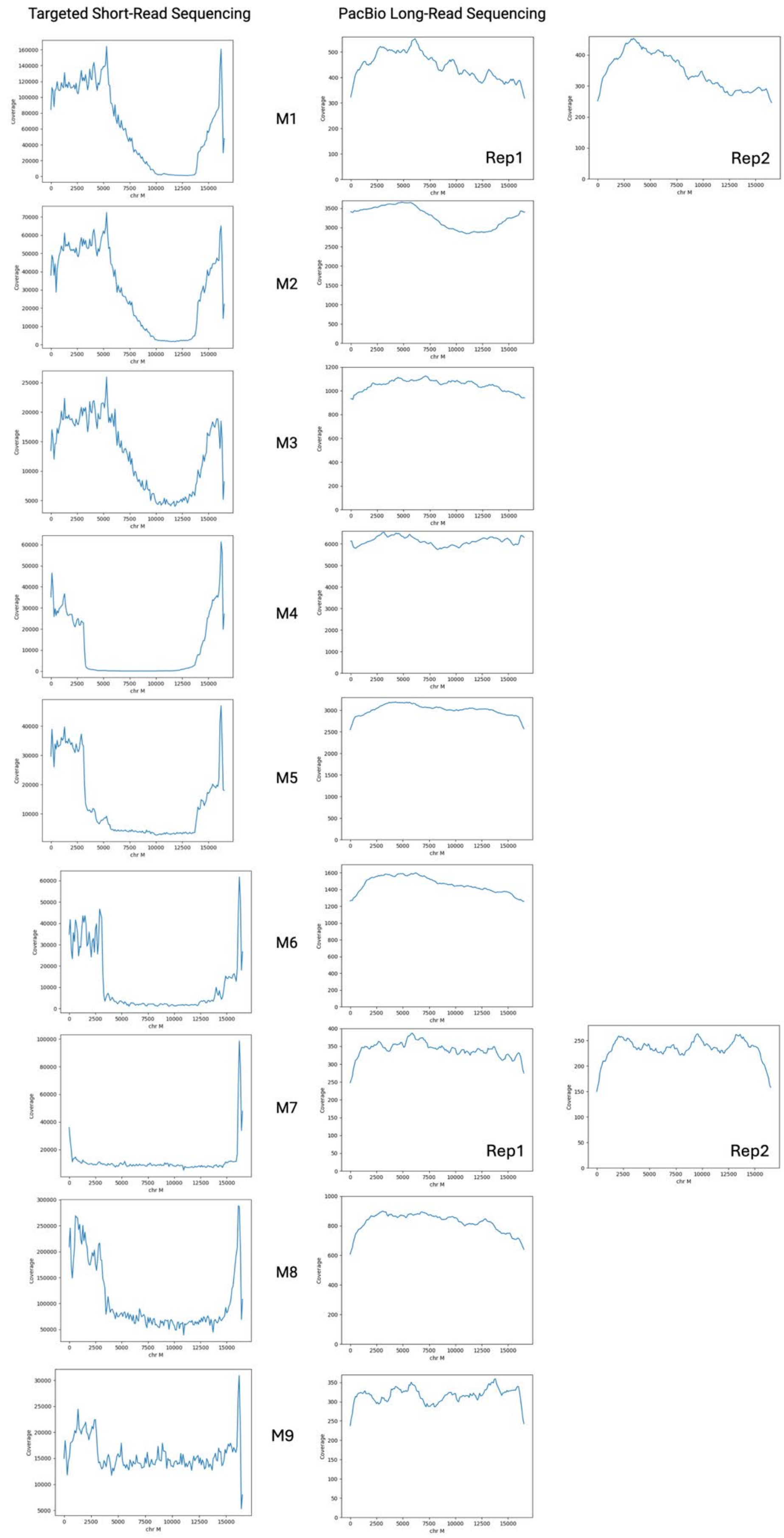

Targeted Short-Read Sequencing
PacBio Long-Read Sequencing
M1
Rep1
Rep2
M2
M3
M4
M5
M6
M7
Rep1
Rep2
M8
M9
Coverage
chr M

This figure plots coverage for SRS and LRS data across the mitogenome for MMD samples. MMD samples don’t show a clear drop in coverage at deletion breakpoints in LRS data due to low heteroplasmy of the MMD. However, they show drops in coverage in the SRS data due to the LR-PCR amplification bias, which overestimates the deletion molecules.

C: Coverage Plots for Control Samples

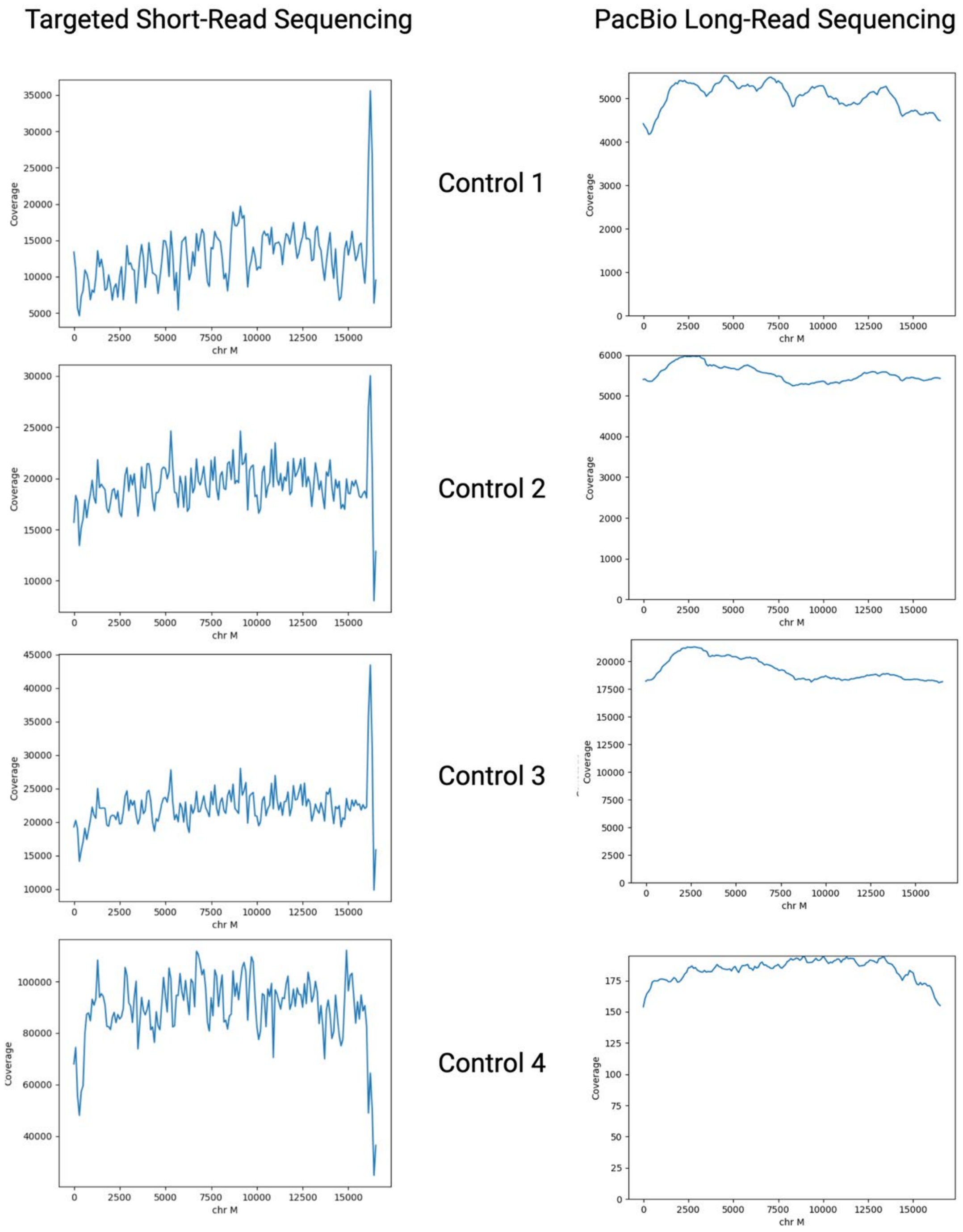

This figure plots coverage for SRS and LRS data across the mitogenome for deletion negative control samples. Control samples show even coverage throughout the mitogenome.

Supplementary Table 1: Haplogroup and coverage analysis

Sample ID	Tissue Type	Haplogroup	Mean nDNA Coverage	Mean mtDNA coverage	Mean Coverage at ND4	Mean Coverage at RNR2
S1	Blood	A2ae	16.33	404.3	219.49	582.03
S2	Muscle	H5c2	1.47	2078.8	1757.85	2330.24
S3	Blood	B4a1a1m1	12.36	265.9	195.81	316.84
S4	Blood	L2c1	16.93	319.3	295.59	344.87
M1 Replicate 1	Muscle	H4a1a	0.22	451.2	411.87	487.52
M1 Replicate 2			0.16	346.2	302.38	414.11
M2	Muscle	K1a4a1a2a	4.8	3266.4	2865.71	3526.36
M3	Muscle	H1as2	1.74	1047.9	1086.67	1104.97
M4	Cardiac Muscle	A2w	0.45	6134.4	6067.22	6299.55
M5	Muscle	H2a2b	1.23	3009.9	3032.07	3032.39
M6	Muscle	J2b1a	2.11	1461.1	1432.25	1549.59
M7 Replicate 1	Muscle	H7a1b	0.18	343.5	334.76	351.83
M7 Replicate 2			0.06	233.8	231.92	252.33
M8	Muscle	T2c1	0.5	822.6	809.07	870.98
M9	Muscle	H1c3a	0.25	316.8	315.04	307.88
Control 1	Muscle	H1c3a	2.22	5058.9	4888.75	5314.47
Control 2	Muscle	T2a1b1a1	6.96	5538.1	5485.23	5925.83
Control 3	Muscle	H5a1g	9.32	19276.7	18428.62	21127.69
Control 4	Blood	A2ah	10.47	183.6	190.96	180.38

Supplementary Table 2: Estimated mtDNA content based on mean coverage of mtDNA and nDNA

	Mean coverage	

**Blood Sample**	**nDNA**	**mtDNA**	**mtDNA content***
S1	16.3	404.3	49.5
S3	12.4	265.9	43.0
S4	16.9	319.3	37.7
Control 4	10.5	183.6	35.1
**Mean**	14.0	293.3	41.8
**Muscle Sample**	**nDNA**	**mtDNA**	**mtDNA content***
S2	1.5	2078.8	2828.3
M1 Replicate 1	0.2	451.2	4101.8
M1 Replicate 2	0.2	346.2	4327.5
M2	4.8	3266.4	1361.0
M3	1.7	1047.9	1204.5
M4	0.5	6134.4	27264.0
M5	1.2	3009.9	4894.1
M6	2.1	1461.1	1384.9
M7 Replicate 1	0.2	343.5	3816.7
M7 Replicate 2	0.1	233.8	7793.3
M8	0.5	822.6	3290.4
M9	0.3	316.8	2534.4
Control 1	2.2	5058.9	4557.6
Control 2	7.0	5538.1	1591.4
Control 3	9.3	19276.7	4136.6
**Mean**	2.1	3292.4	3118.8

*mtDNA content=(mtDNA/nDNA)x2

Supplementary Table 3: Reproducibility of SNV detection by LRS

SNVs detected in M1		SNVs detected in M7	
M1 Replicate 1	M1 Replicate 2	M7 Replicate 1	M7 Replicate 2
m.263A>G	m.263A>G	m.263A>G	m.263A>G
m.750A>G	m.750A>G	m.750A>G	m.750A>G
m.1438A>G	m.1438A>G	m.1393G>A	m.1393G>A
m.3992C>T	m.3992C>T	m.1438A>G	m.1438A>G
m.4024A>G	m.4024A>G	m.1719G>A	m.1719G>A
m.4769A>G	m.4769A>G	m.4769A>G	m.4769A>G
m.5004T>C	m.5004T>C	m.4793A>G	m.4793A>G
m.8269G>A	m.8269G>A	m.8860A>G	m.8860A>G
m.8860A>G	m.8860A>G	m.10931T>C	m.10931T>C
m.9123G>A	m.9123G>A	m.15326A>G	m.15326A>G
m.10044A>G	m.10044A>G	m.16261C>T	m.16261C>T
m.14365C>T	m.14365C>T	m.16519T>C	m.16519T>C
m.14582A>G	m.14582A>G		
m.15326A>G	m.15326A>G		
